# HIV-1-infection in a man who has sex with men despite self-reported excellent adherence to pre-exposure prophylaxis, the Netherlands, August 2021: be alert to emtricitabine/tenofovir-resistant strain transmission

**DOI:** 10.2807/1560-7917.ES.2022.27.14.2200275

**Published:** 2022-04-07

**Authors:** Jeffrey CD Koole, Feline de la Court, Matthijs RA Welkers, Kenneth Yap, Janneke E Stalenhoef, Suzanne Jurriaans, Henry JC de Vries, Eline LM Op de Coul, Maria Prins, Elske Hoornenborg

**Affiliations:** 1 Department of Infectious Diseases, Public Health Service Amsterdam, Amsterdam, the Netherlands; 2 Department of Medical Microbiology and Infection Prevention, Amsterdam UMC, Amsterdam, the Netherlands; 3 Department of Internal Medicine, OLVG, Amsterdam, the Netherlands; 4 Department of Dermatology, Amsterdam Institute for Infection and Immunity (AII), Amsterdam UMC, University of Amsterdam, location Academic Medical Centre, Amsterdam, the Netherlands; 5 Centre for Infectious Disease Control, National Institute for Public Health and the Environment (RIVM), Bilthoven, the Netherlands; 6 Department of Infectious Diseases, Amsterdam Infection and Immunity Institute (AII), Amsterdam UMC, University of Amsterdam, Amsterdam, the Netherlands

**Keywords:** case report, HIV pre-exposure prophylaxis, HIV, antiretroviral drug resistance, genomic surveillance, prevention, diagnostics

## Abstract

In August 2021, a man who has sex with men was diagnosed with HIV-1 infection despite using event-driven pre-exposure prophylaxis for over 2 years with self-reported excellent adherence. Sequencing identified resistance-associated mutations (RAM) M184V and K65R, conferring resistance to emtricitabine and tenofovir, and RAM V108I and E138A. Background RAM prevalence was two of 164 (1.2%) new HIV diagnoses in Amsterdam (2017–19). We reiterate the need for frequent HIV testing among PrEP users and additional testing in case of symptoms.

Both daily and event-driven HIV pre-exposure prophylaxis (PrEP) regimens are safe and effective biomedical prevention strategies against HIV-1 infection [1,2]. However, PrEP failure can occur, especially when individuals are exposed to an HIV-1 strain resistant to emtricitabine (FTC) and/or tenofovir disoproxil fumarate (TDF) [3-7] or, rarely, wild-type HIV-1 [8]. In the Netherlands, PrEP became available via a demonstration project followed by a national PrEP pilot (NPP) for a maximum of 8,500 persons in 2019 [9,10]. We describe a case of PrEP failure in a person who self-reported excellent adherence, probably via a sexually acquired FTC/TDF-resistant HIV strain, although the case occurred in an area with a very low background prevalence of HIV resistance.

## Pre-exposure prophylaxis care in the Netherlands

During quarterly PrEP consultations and following national guidelines [11], nucleic acid amplification tests on self-collected urine samples, oropharyngeal and anal swabs are used to detect *Neisseria gonorrhoeae* and/or *Chlamydia trachomatis* infections. *Treponema pallidum* particle agglutination and venereal disease research laboratory assays on serum are used to detect syphilis. We screen for HIV-1 infection using a combined fourth generation HIV antigen/antibody test (in our laboratory: Liason XL Murex HIV Ag/Ab) on serum. This is followed by a Western blot, p24 antigen test and a test for HIV-1 RNA load on serum if the combined HIV Ag/Ab test is positive or indeterminate. In addition, we counsel PrEP users on PrEP adherence and on how to safely start, stop and switch between daily and event-driven PrEP.

## Case description

In August 2021, a man who has sex with men (MSM) in his 30s was diagnosed with a primary HIV-1 infection despite having used event-driven PrEP (consisting of FTC/TDF) for more than 2 years with self-reported excellent adherence, defined as 100% correct use according to the event-driven regimen. His medical history revealed multiple sexually transmitted infections (STI) in the previous years (2014–21), including syphilis stage I, scabies, multiple infections with *N. gonorrhoea* and *C. trachomatis*, one of which was with a serovar causing lymphogranuloma venereum. The patient started PrEP in 2019 at the centre of sexual health (CSH) of the Public Health Service Amsterdam, reported consistent and correct use of event-driven PrEP during all condomless anal sex acts and claimed to be well acquainted with the PrEP intake regimen (two tablets 2–24 h before the sex act, followed by one tablet 24 h and 48 h later) [2]. Moreover, he reported that he had always taken an additional tablet 72 h after the first tablets for extra protection, although he knew that this extra tablet was not necessary.

The Figure visualises the timeline of PrEP use, sexual behaviour, symptoms and clinical events between the last negative HIV test and antiretroviral treatment (ART) initiation. During the patient's most recent PrEP visit at the CSH before HIV diagnosis, all HIV tests (including HIV RNA determined retrospectively on a stored sample) and STI test results had been negative. Seven weeks later, in-between the scheduled quarterly PrEP visits, he returned to the clinic because of symptoms including loss of 6% of body weight, diarrhoea, oropharyngeal mucosal defects and myalgia. At this visit, the patient was diagnosed with HIV infection Fiebig stage IV. In the 7 weeks between his last negative HIV test result and HIV diagnosis, he reported receptive condomless anal sex with four male partners (no group sex), both in the Netherlands and in Belgium, and sexualised drug use (gamma-hydroxybutyric acid, poppers, ecstasy and kamagra; no injecting drug use). His recent sex partners were notified of possible exposure to HIV and were advised to get tested.

**Figure:**
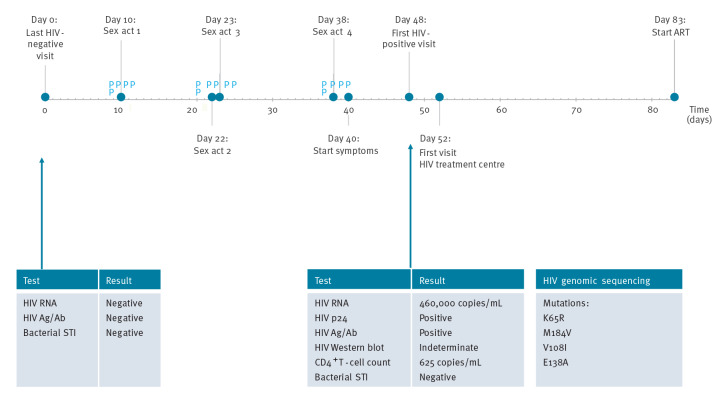
Timeline of reported sex acts, healthcare events from last negative HIV-test until antiretroviral therapy initiation, and PrEP failure in a man who has sex with men with self-reported excellent PrEP adherence, the Netherlands, August 2021

Ab: antibody; Ag: antigen; ART: antiretroviral therapy; cop: copies; p: PrEP tablet taken (self-reported); STI: sexually transmitted infections.

Sanger sequencing identified resistance-associated mutations (RAM) M184V and K65R conferring resistance to both FTC and TDF, and V108I and E138A conferring resistance to nevirapine (NVP) and rilpivirine (RPV). No RAM conferring resistance to integrase or protease inhibitors were identified. The patient initiated ART, consisting of dolutegravir and darunavir/cobicistat, 35 days after his first diagnosis, when the genomic resistance results were reported.

## Discussion

We describe a primary HIV-1 infection with a drug-resistant strain in an MSM on PrEP who reported excellent adherence to the event-driven regimen for more than 2 years. HIV genomic sequencing identified several RAM associated with resistance to FTC and TDF as well as NVP and RPV.

Possible explanations for the PrEP failure in our case include: (i) HIV infection by transmission of a strain with several RAM (M184V, K65R, V108I and E138A); (ii) HIV infection with a strain with the RAM V108I and E138A, followed by the acquisition of RAM M184V and K65R under pressure of FTC and TDF; (iii) HIV infection with wild-type genotype, followed by development of the identified RAM during PrEP use; (iv) incorrect adherence resulting in low or no protective drug levels during exposure to an HIV strain with multiple RAM. The combination of the identified RAM makes the first explanation most likely. Although theoretically possible, it is unlikely that the short-term and limited drug exposure was sufficient to select for both M184V and K65R, with only few M184V mutations, and even more rarely K65R, described in the literature [12]. We hypothesise that the identified viral strain was sexually acquired from a treatment-experienced individual with detectable HIV viral load and multiple RAM, probably as a consequence of non-adherence to ART.

A combination of RAM similar to those found in this case (M184V, K65R, V108I and E138A) has not been reported in PrEP users. However, six cases of HIV infection despite PrEP use have been described previously [3-5,8]; in two cases both M184V and K65R were identified [6,7].

The Amsterdam University Medical Centres (Amsterdam UMC) determined the baseline reverse transcriptase resistance between 2017 and 2019 among 164 individuals recently diagnosed with HIV infection. They identified the RAM M184V in only two individuals (1.2%) (data not shown). No other RAM to TDF and/or FTC were identified. This indicates a very low risk of transmitting resistance to PrEP users in this region. Nonetheless, in the upcoming years, rare breakthrough infections with strains containing RAM to FTC and/or TDF may occur more often, related to the expected increase in PrEP use. This highlights the importance of genomic sequencing of strains identified in individuals recently diagnosed with HIV infection, especially among those with recent PrEP use, for early detection of drug-resistant strains in order to prevent further onward transmission and to guide the choice of a suitable ART regimen [13].

Unfortunately, we could not verify the self-reported PrEP adherence in this case. An objective method to assess adherence in daily PrEP is measuring intracellular tenofovir levels in dried blood spots [14]. However, this is not applicable to event-driven PrEP use as the levels will not provide information on whether the PrEP tablets were correctly used before and after sexual contact [15].

Although symptoms are not commonly reported in case of an HIV infection during PrEP use, the HIV infection in our case was diagnosed during an additional PrEP care visit because of symptoms related to acute HIV infection [16]. Therefore, we recommend additional low-threshold HIV testing using a test with a short window period, such as fourth generation HIV antigen/antibody test or an HIV RNA test, in between scheduled PrEP follow-up visits in case of symptoms. Furthermore, combined HIV prevention strategies remain important, including condom use and counselling about risk reduction. Lastly, prompt ART initiation after diagnosis and optimising ART adherence to achieve and sustain undetectable HIV viral load are key to preventing ongoing HIV transmission [17].

## Conclusion

Acquisition of an HIV-1 strain with RAM can occur among PrEP users with excellent adherence. We suggest that the most likely underlying cause in this case was sexual acquisition of a TDF/FTC-resistant strain from an individual on ART who was not virally suppressed at the time of HIV transmission. It is thus important to frequently test PrEP users for HIV, regardless of the background prevalence of resistance in newly diagnosed HIV, reported adherence and especially in case of symptoms related to acute HIV infection. Finally, promoting adherence to ART in HIV-positive individuals remains important to prevent onward transmission.
